# Comparative analysis of female breast cancer burden in China along the global sociodemographic gradient: risk attribution, decomposition, and age-period-cohort evidence from GBD 2021

**DOI:** 10.1016/j.clinsp.2026.101166

**Published:** 2026-09-30

**Authors:** Yixin Si, Jialin Liu, Liuqiyu Xu, Wenya Ye, Jiating Li, Yun Peng, Zhen Hu

**Affiliations:** a Department of Ultrasound, The Second Affiliated Hospital, Jiangxi Medical College, Nanchang University, Nanchang, 330006, China; b Department of Radiology, The Second Affiliated Hospital, Jiangxi Medical College, Nanchang University, Nanchang, 330006, China

**Keywords:** Breast cancer, Global disease burden, Population Attributable Fraction, Four-effect decomposition, Age-period-cohort model

## Abstract

**Objective:**

To assess temporal changes in female breast cancer burden in China from 1990 to 2021, its global and Socio-Demographic Index (SDI) status, attributable risks, death drivers and age-period-cohort (APC) features based on GBD 2021 data.

**Methods:**

We extracted data on incidence, deaths, DALYs and seven attributable risks for China, the world and five SDI regions. Estimated Annual Percentage Change (EAPC) analyzed age-standardized rate trends. Shapley decomposition divided attributable death changes into population growth, aging, age-specific mortality and Population Attributable Fraction (PAF). APC models estimated temporal drifts.

**Results:**

From 1990 to 2021, the Age-Standardized Incidence Rate (ASIR) increased from 24.54 to 29.36 per 100,000 (EAPC = 0.30%, 95% CI: 0.20 to 0.41%) in China, whereas the Age-Standardized Mortality Rate (ASMR) decreased from 10.65 to 6.46 per 100,000 (EAPC = −2.02%, 95% CI −2.20 to −1.85%). By 2021, both rates were lower in China than in the global population and all five SDI regions. The leading PAFs for Disability-Adjusted Life-Years (DALYs) burden were high red meat intake, secondhand smoke, high fasting plasma glucose, high body mass index, and low physical activity. Decomposition showed that increasing risk-specific attributable deaths were mainly driven by population growth and ageing, while declining age-specific mortality contributed negatively. Incidence net drift was 0.232%/year; mortality net drift was −2.115%/year.

**Conclusion:**

China showed a transitional breast cancer burden pattern, characterized by slowly rising incidence, declining age-standardized mortality, and a shift toward metabolic risk contribution. Population ageing and metabolic risk accumulation may continue to increase the absolute burden despite improvements in mortality risk.

## Highlights

• Incidence rose slightly, while mortality declined markedly in China.
• China had lower 2021 incidence and mortality than all SDI regions.
• High BMI and fasting glucose reflected a growing metabolic risk shift.
• Four-effect decomposition showed ageing and growth drove attributable deaths.
• APC analysis showed slight incidence growth but sustained mortality decline.

## Introduction

Breast cancer ranks among the most prevalent malignancies affecting women worldwide and represents a significant contributor to cancer-related mortality and disability burden among females.^1^^,^^2^ Despite global increases in female breast cancer cases attributable to population growth, aging, and changing risk factor exposures, significant heterogeneity exists in incidence and mortality burdens across regions with varying levels of the Socio-Demographic Index (SDI).^3^^,^^4^ In this global context, China’s female breast cancer burden holds particular public health significance. China’s large female population base and accelerating aging process mean that even modest changes in breast cancer incidence or mortality rates translate into substantial absolute case numbers and healthcare demands. China differs from typical high-income countries and most low SDI regions in risk factor profiles, screening accessibility, diagnostic capacity, and healthcare resource allocation. Comparing China with global patterns and across SDI levels helps clarify its epidemiological position and prevention priorities.

Previous GBD-based studies have progressively characterized the changing burden of female breast cancer at the global and national levels. Global analyses have documented substantial temporal variations in incidence, mortality, and Disability-Adjusted Life Years (DALYs), as well as marked differences across SDI quintiles.^5^, ^6^, ^7^ Against this broader epidemiological backdrop, studies focusing on China have shown distinct long-term changes in breast cancer burden and have compared these trends with those observed globally or in selected comparator countries.^8^, ^9^, ^10^ Beyond aggregate burden estimates, increasing attention has been given to attributable risk factors, with metabolic risks, particularly high body mass index (BMI) and high fasting plasma glucose, accounting for an increasing proportion of breast cancer burden among Chinese women.^11^, ^12^, ^13^ Age-Period-Cohort (APC) analyses have further shown substantial age and cohort heterogeneity in breast cancer mortality and risk-attributable mortality.^12^^,^^14^ However, existing studies have primarily described temporal changes in the overall breast cancer burden and risk-attributable burden, whereas the extent to which different factors contribute to changes in risk-specific attributable deaths remains insufficiently quantified. In particular, temporal changes in deaths attributable to individual risk factors may reflect the combined effects of population growth, changes in population age structure, changes in age-specific breast cancer mortality rates, and changes in the Population Attributable Fraction (PAF), and the respective contributions of these components require further quantification within a unified framework.

Therefore, using GBD 2021 data from 1990 to 2021, we employed a four-component Shapley decomposition framework to decompose risk-specific attributable breast cancer deaths, and separately quantified the contributions of population growth, age structure shifts, age-specific mortality changes, and PAF variations.^15^, ^16^, ^17^ We also assessed female breast cancer burden, major attributable risks, and APC patterns in China to further clarify the major drivers and their relative contributions to changes in risk-specific attributable breast cancer deaths.

## Materials and methods

### Data collection

This study analyzed publicly available aggregated data from GBD 2021. GBD 2021 systematically estimated disease burden, risk factor exposures, and attributable burdens across 204 countries and territories from 1990 to 2021, providing a unified analytical framework for comparisons across countries and SDI levels.^18^

This study extracted female breast cancer-related data for China, global estimates, and the five SDI quintiles from 1990 to 2021. Primary metrics included incidence count, death count, DALYs, Age-Standardized Incidence Rate (ASIR), Age-Standardized Mortality Rate (ASMR), Age-Standardized DALY Rate (ASDR), and their corresponding 95% Uncertainty Intervals (95% UIs). Age-specific analyses began with the 15‒19 age group as the starting age group, followed by 5-year age increments, culminating with the 95-years and above group as the oldest age group. As this study used publicly released aggregated data at regional and age-group levels without involving individually identifiable information or human/animal experimentation, additional ethical approvals and informed consent were not required.

### Attributable risk factor definitions

Female breast cancer risk factors were determined based on the GBD 2021 Cancer Attributable Risk framework. This study included seven risk factors from GBD 2021 associated with female breast cancer based on sufficient or suggestive evidence and available population exposure distribution data, including smoking, high alcohol use, secondhand smoke exposure, low physical activity, high red meat consumption, high BMI, and high fasting plasma glucose.^5^ According to GBD 2021 classification, smoking, secondhand smoke exposure, high alcohol use, low physical activity, and high red meat consumption are categorized as behavioral risks; high BMI and high fasting plasma glucose as metabolic risks. The exposure thresholds, theoretical minimum risk exposure levels, and attribution estimation methods for all risk factors were defined according to the GBD 2021 standard.^3^ The PAFs, stratified by location, year, age, and sex, to quantify the female breast cancer deaths and DALYs attributable to corresponding risk factors were extracted.

### Temporal trend analysis

The Estimated Annual Percentage Change (EAPC) was used to quantify temporal trends in ASIR and ASMR from 1990 to 2021. A linear regression model was fitted with the natural logarithm of the age-standardized rate as the dependent variable and year as the independent variable:ln(rate)=α+β×year. The EAPC was calculated as $EAPC=100\;\times (e^{\beta }-1)$. An EAPC greater than 0 indicates an overall increase in the indicator, while an EAPC less than 0 indicates an overall decrease. The 95% Confidence Interval (95% CI) for the EAPC was estimated based on the regression model.^19^

### Four-effect decomposition of attributable death changes

To assess the sources of change in attributable deaths, this study decomposed the change in female breast cancer attributable deaths from 1990 to 2021 into four components: Population growth (P), age-Structure change (S), cause-specific mortality Rate change (R), and PAF change (F).^15^ Attributable deaths for each age group were calculated as: $AD=\sum_{a}Pop_{a}\times R_{a}\times F_{a}$; where AD represents attributable deaths; Pop_a_ denotes the population of age group a; R_a_ indicates the age-specific mortality rate, and F_a_ refers to the age-specific PAF. By defining Sa as the proportion of age group a within the total population ($S_{a}=Pop_{a}/P)$, attributable deaths can be expressed as: $AD=P\times \sum_{a}\left(S_{a}\times R_{a}\times F_{a}\right),$ where P represents total population size. We employed the Shapley decomposition approach to decompose changes in attributable deaths from 1990 to 2021, quantifying the individual contributions of the four aforementioned components to these variations. The Shapley decomposition was implemented using a custom Python script. For each location and risk factor, marginal contributions were calculated across all possible permutations of the four components (4! = 24 permutations). The average marginal contribution across permutations was considered the contribution of each component, thereby avoiding dependence on a single substitution order.^16^^,^^17^ Results were presented as absolute contributions. For visualization of the relative contribution structure, signed component contributions were additionally normalized by the sum of the absolute values of all four component contributions (Σ|contribution| = 100%), while retaining the direction of each contribution. Internal consistency of the decomposition was verified by confirming that the sum of the four Shapley component contributions reproduced the observed change in attributable deaths, with only negligible numerical error.

### APC analysis

An APC model was applied to evaluate age, period, and birth cohort effects in long-term trends of female breast cancer incidence and mortality rates. APC analyses were performed using R software (version 4.4.1) with the Epi package (version 2.60) and the apc. fit function. The APC model was fitted using a Poisson regression framework with the logarithm of population size included as an offset term: $\log\left(E[D_{ijk}]\right)=\log\left(Pop_{ijk}\right)+\mu +\alpha_{i}+\beta_{j}+\gamma_{k}$, where D_ijk_ represents the number of incidence or mortality events in the i-th age group, j-th period, and k-th birth cohort; $\log(Pop_{ijk})$ denotes the corresponding person-years or population exposure; μ is the intercept; and α_i_, β_j_, and γ_k_ represent the age, period, and cohort effects, respectively. Because age, period, and cohort are linearly dependent (cohort = period-age), APC effects were estimated using the APC model parameterization implemented in the Epi package rather than the intrinsic estimator approach.^20^^,^^21^ Age groups were categorized into closed 5-year intervals from 15‒19 to 90‒94 years; the open-ended 95+ age group was excluded from APC modelling. Calendar years were grouped into six complete 5-year periods from 1990‒1994 through 2015‒2019; the incomplete 2020‒2021 interval was excluded to preserve a regular age-period lattice. Birth cohorts were derived based on the difference between calendar period and age. Relative Risks (RRs) and corresponding 95% confidence intervals were calculated using reference categories of 50–54 years for age, 2005‒2009 for calendar period, and the 1965 birth cohort. Net drift and local drift were estimated from the fitted Poisson APC model. Net drift represented the overall annual percentage change during the study period, whereas local drift represented age-specific annual percentage changes estimated from age-by-period interaction models.^22^ Model convergence and goodness-of-fit were assessed using residual deviance, degrees of freedom, deviance-to-degrees-of-freedom ratios, Pearson chi-square statistics, and AIC. As a sensitivity analysis, the APC analysis was repeated after restricting the age range to 20‒89 years.

## Results

### Long-term trends in female breast cancer burden in china across SDI gradients

From 1990 to 2021, the ASIR for female breast cancer exhibited a mild upward trend in China, while the ASMR showed a consistent decline. Specifically, the ASIR increased from 24.54 per 100,000 to 29.36 per 100,000, with an EAPC of 0.30% (95% CI: 0.20 to 0.41%). The ASMR declined from 10.65 per 100,000 to 6.46 per 100,000, with an EAPC of −2.02% (95% CI: −2.20 to −1.85%) (Table 1). Compared with different SDI tiers, the ASIR in 1990 in China exceeded those in upper-middle, middle, lower-middle, and low-SDI regions but was below the global average (42.37/100,000) and high-SDI regions (60.99/100,000). The ASMR in China was lower than the global average and all SDI tiers. By 2021, both ASIR and ASMR in China were lower than the global average and all five SDI tiers. From 1990 to 2021, the growth in ASIR in China was less pronounced than in middle-SDI regions (EAPC = 2.17%), lower-middle-SDI regions (EAPC = 2.50%), and low-SDI regions (EAPC = 2.10%). Conversely, China demonstrated the fastest decline in ASMR among all comparison regions, exceeding the global rate (EAPC = −0.54%) and that of high-SDI regions (EAPC = −1.40%) (Fig. 1).

**Table 1:** Trends in ASIR and ASMR of female breast cancer in 1990 and 2021 across China, global and SDI regions.

Region	ASIR			ASMR		
	1990	2021	EAPC (95% CI)	1990	2021	EAPC (95% CI)
Global	42.37	47.03	0.15 (0.10 to 0.21)	17.04	15.44	−0.54 (−0.63 to −0.46)
High SDI	60.99	59.58	−0.18(−0.29 to −0.07)	20.82	14.18	−1.40 (−1.47 to −1.33)
High-middle SDI	24.04	33.69	0.84 (0.77 to 0.91)	12.17	11.55	−0.42 (−0.50 to −0.35)
Middle SDI	18.82	37.76	2.17 (2.10 to 2.24)	10.87	16.13	1.20 (1.15 to 1.24)
Low-middle SDI	16.41	36.55	2.50 (2.28 to 2.73)	10.79	19.13	1.77 (1.56 to 1.99)
Low SDI	16.03	32.81	2.10 (1.81 to 2.40)	11.09	18.37	1.46 (1.23 to 1.70)
China	24.54	29.36	0.30 (0.20 to 0.41)	10.65	6.46	−2.02 (−2.20 to −1.85)

Note: Rates are per 100,000 population. ASIR, Age-Standardized Incidence Rate; ASMR, Age-Standardized Mortality Rate; SDI, Socio-Demographic Index; EAPC, Estimated Annual Percentage Change; CI, Confidence Interval.

**Fig. 1:**
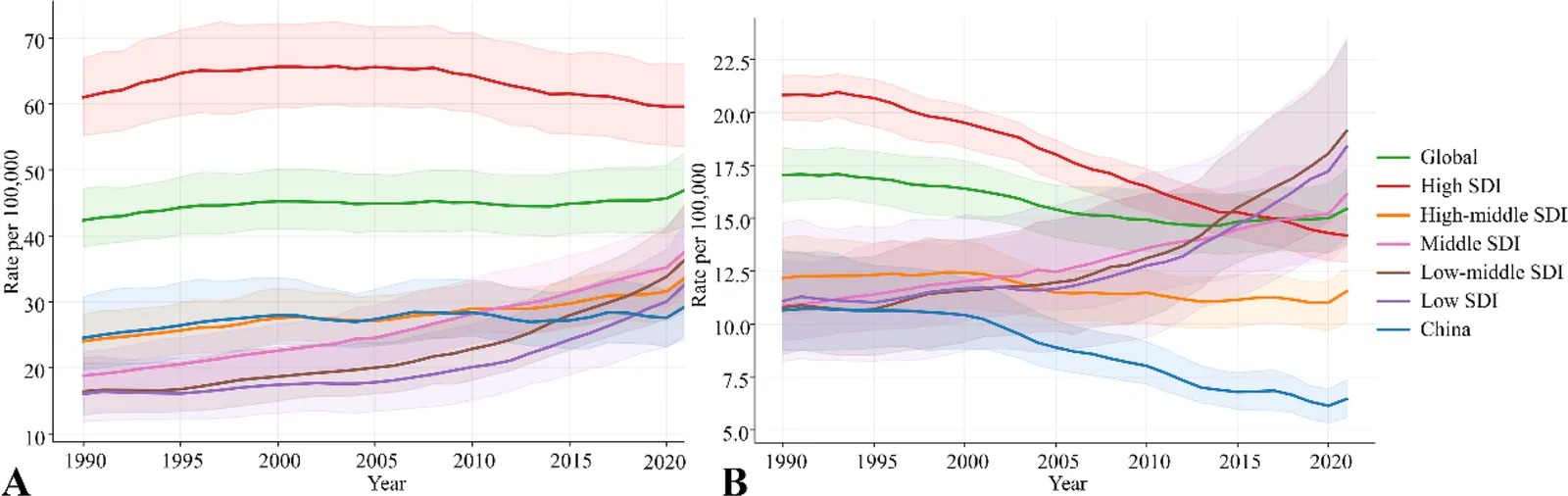
Temporal trends in age-standardized female breast cancer burden across China, the World, and five SDI quintiles, 1990‒2021: (A) ASIR; (B) ASMR.

### Evolution of the major attribution risk spectrum for female breast cancer in China

The ranking of major attributable risks for female breast cancer in China remained generally stable from 1990 to 2021. Using PAF for DALYs burden as an example, the top five risk factors were consistently high red meat consumption, secondhand smoke exposure, high fasting plasma glucose, high BMI, and low physical activity. Only high alcohol use and smoking changed their relative ranking. Among these risks, the PAF for high BMI rose from 2.6 to 4.3%, showing the most pronounced increase, whereas the PAF for smoking decreased from 1.2 to 0.7% (Fig. 2).

**Fig. 2:**
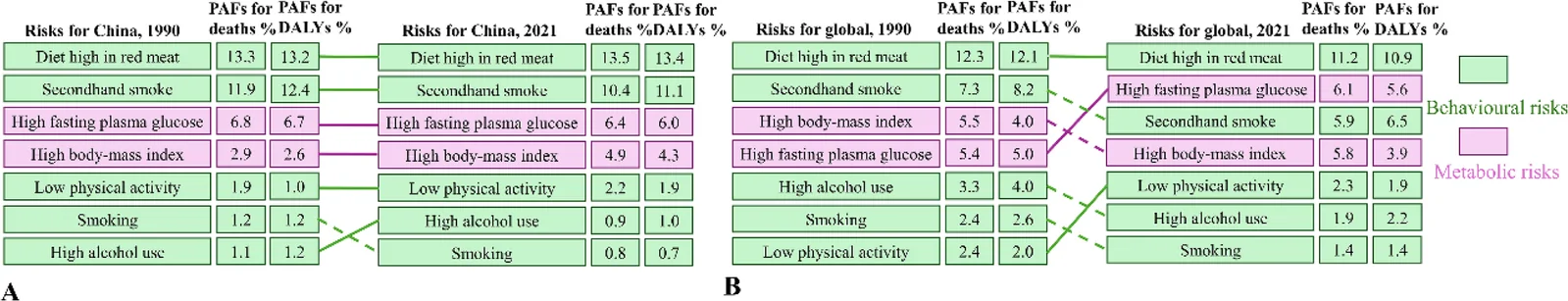
Changes in the PAF values and rankings of attributable risk factors for female breast cancer in China and globally in 1990 versus 2021 (A) China (B) Global.

Supplementary Fig. S1 shows changes in PAF rankings across different SDI levels. In 2021, China’s PAF for high red meat consumption was 13.4%, exceeding the global average (10.9%) and all SDI levels, and was comparable to high-SDI regions (13.3%) but markedly higher than low SDI regions (8.3%). The PAF for secondhand smoke exposure was 11.1%, also higher than both the global average and high-SDI regions. For metabolic risks, the PAF for high BMI in China was 4.3%, which was lower than that in high-SDI regions (6.3%) but higher than that in middle-SDI regions (3.6%). The PAF for high fasting plasma glucose was 6.0%, higher than that in low-SDI and low-middle-SDI regions and close to levels observed in higher SDI tiers. In contrast, the PAFs for smoking and high alcohol use in China were both lower than the global average and those in high-SDI regions.

### Four-effect decomposition results of attributable mortality change

Table 2, Fig. 3 and Supplementary Table S1 present the four-effect decomposition results. From 1990 to 2021, the arithmetic sum of risk-specific attributable deaths across the seven selected GBD risk factors increased from 193,166.0 to 277,569.7, representing an increase of 84,403.7. These aggregated values should not be interpreted as a joint attributable burden because risk-specific attributable deaths are not mutually exclusive across risk factors. Four-effect decomposition revealed that population growth and age-structure change were the major positive contributors, contributing 127,560 deaths (151.1% of the arithmetic increase) and 81,550 deaths (96.6%), respectively. Conversely, changes in age-specific mortality rates contributed −123,380 deaths (−146.2%), partially offsetting the increase. The contribution of PAF changes was relatively small, accounting for −1320 deaths (−1.6%).

**Table 2:** Four-effect decomposition of changes in female breast cancer deaths attributable to selected risk factors in China and globally, 1990‒2021.

Location	Risk	Δ Attributable deaths	Population growth contribution	Age-structure contribution	Age-specific mortality rate contribution	PAF contribution
China	Diet high in red meat	29203.52	44770.37	25538.31	−42244.1	1138.975
	High fasting plasma glucose	12597.92	22124.05	13739.1	−20252.2	−3013.06
	Secondhand smoke	12600.47	37646.92	19332.69	−36814.5	−7564.65
	High body-mass index	22719.72	10197.16	13519.34	−12284	11287.19
	Low physical activity	6728.455	6797.69	4991.781	−5572.97	511.958
	High alcohol use	308.936	1870.333	2623.333	−3411.21	−773.524
	Smoking	244.7171	4148.498	1802.696	−2798.45	−2908.03
Global	Diet high in red meat	339505.3	357808.6	106745	−65477.8	−59570.6
	High fasting plasma glucose	241909.1	175840.4	57470.82	−37425.8	46023.81
	Secondhand smoke	141056.8	201854.1	46832.69	−30286	−77343.9
	High body-mass index	232051.5	158697.1	90153.15	−56589.6	39790.89
	Low physical activity	81600.75	71161.05	26286.18	−15627	−219.487
	High alcohol use	8058.182	66612.28	30044.11	−11792.9	−76805.3
	Smoking	7677.94	63710.24	10015.14	−11511.9	−54535.6

Note: Values in the four-effect decomposition columns represent the absolute contribution of each component to the change in risk-specific attributable deaths between 1990 and 2021. PAF, Population Attributable Fraction. Risk-specific attributable deaths are not mutually exclusive across risk factors and should be interpreted as risk-specific attributable burden rather than additive individual-level cases.

**Fig. 3:**
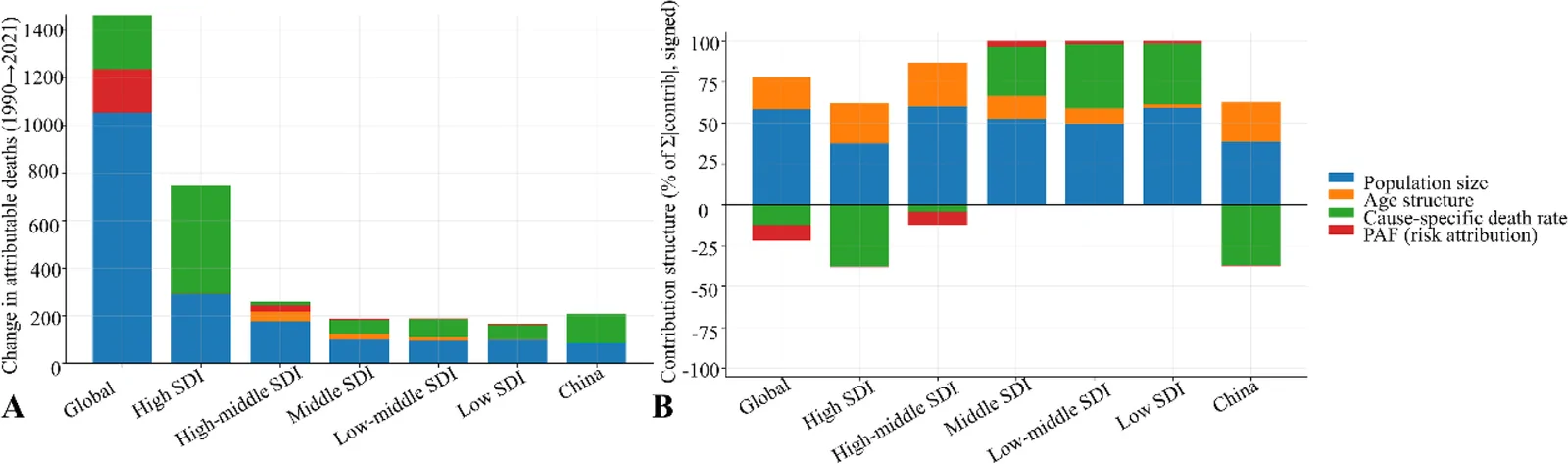
Four-factor Shapley decomposition of changes in female breast cancer risk-specific attributable deaths, 1990‒2021. (A) Absolute contributions. (B) Signed contribution composition normalized to Σ|contribution| = 100%. Values aggregated across the seven risks represent arithmetic sums rather than a joint attributable burden.

Globally, the increase in attributable deaths was primarily driven by population growth, whereas changes in age-specific mortality rate exerted a negative contribution. The negative contribution of age-specific mortality rate changes was more pronounced in high-SDI regions. In low- and middle-SDI regions, population growth, age-structure change, and PAF changes jointly contributed to increases in attributable deaths. In contrast, China’s main pattern was that population growth and aging jointly contributed to the increase in absolute attributable deaths, while the decline in age-specific mortality rate partially offset this increase (Fig. 3). The driving patterns differed across risk factors. High red meat consumption contributed to an increase of 29,203.5 attributable deaths, primarily driven by population growth and age-structure changes. High BMI contributed to an increase of 22,719.7 attributable deaths, with PAF changes accounting for 11,287.2 cases (49.7%). Although secondhand smoke exposure and high fasting plasma glucose remained major attributable risks, PAF changes contributed −7564.7 cases (−60.0%) and −3013.1 cases (−23.9%), respectively.

### Age, period, and cohort effects

The APC model demonstrated that the age effect on female breast cancer mortality in China increased with advancing age and accelerated further in advanced age groups. The mortality age-effect curves remained relatively flat globally and in high-, high-middle-, and middle-SDI regions. By contrast, they exhibited more pronounced increases after age 70 in low-middle- and low-SDI regions. China’s age-effect pattern was intermediate between those of high-SDI and low-middle-SDI regions.

The period effect showed that the incidence RR in China remained close to the reference level across calendar periods, whereas the mortality RR declined progressively from 1990‒1994 to 2015‒2019. Across SDI regions, incidence period effects showed heterogeneous patterns, with increases in low- and low-middle-SDI regions and relatively limited changes in China and high-SDI regions. For mortality, China and high-SDI regions showed declining period effects, whereas low- and low-middle-SDI regions showed increasing trends. The cohort effect in China increased toward the 1960‒1965 birth cohorts and subsequently declined in later cohorts for both incidence and mortality. Cohort patterns also differed across SDI regions, indicating substantial heterogeneity in generational trends.

The net drift in incidence of female breast cancer in China was 0.232% per year (95% CI: 0.222 to 0.242%), and the net drift in mortality was −2.115% per year (95% CI: −2.132 to −2.099%).This pattern aligns with the sustained mortality decline observed globally and in high-SDI regions, although China’s incidence net drift remained slightly positive. In contrast, middle, low-middle, and low SDI regions exhibited positive net drifts in both incidence and mortality, indicating increasing temporal trends in these areas (Table 3).

**Table 3:** Net drift of female breast cancer incidence and mortality by location, 1990‒2019.

Location	Incidence Net Drift (%)	Deaths Net Drift (%)
China	0.232 (0.222‒0.242)	−2.115 (−2.132‒−2.099)
Global	0.109 (0.105‒0.112)	−0.618 (−0.624‒−0.612)
High SDI	−0.143 (−0.147‒−0.139)	−1.365 (−1.373‒−1.358)
High-middle SDI	0.751 (0.741‒0.761)	−0.542 (−0.558‒−0.527)
Middle SDI	2.199 (2.183‒2.214)	1.214 (1.192‒1.237)
Low-middle SDI	2.563 (2.547‒2.579)	1.750 (1.728‒1.771)
Low SDI	2.142 (2.125‒2.159)	1.439 (1.418‒1.461)

Supplementary Table S2 presents local drift results, revealing significant age heterogeneity in breast cancer incidence trends. The local drift in breast cancer incidence among Chinese women shifted from negative to positive around age 45‒49, peaking in the 85‒89 age group (1.715% per year). Mortality local drift remained negative across all age groups, with the steepest decline (−5.710% per year) occurring in the 20‒24 age group. High-SDI regions exhibited negative local drift for both incidence and mortality in most age groups. Conversely, low-middle- and low-SDI regions displayed positive local drift across nearly all age groups, most notably in younger and middle-aged groups (Fig. 4). Sensitivity analyses restricted to women aged 20‒89 years yielded materially similar APC and drift patterns.

**Fig. 4:**
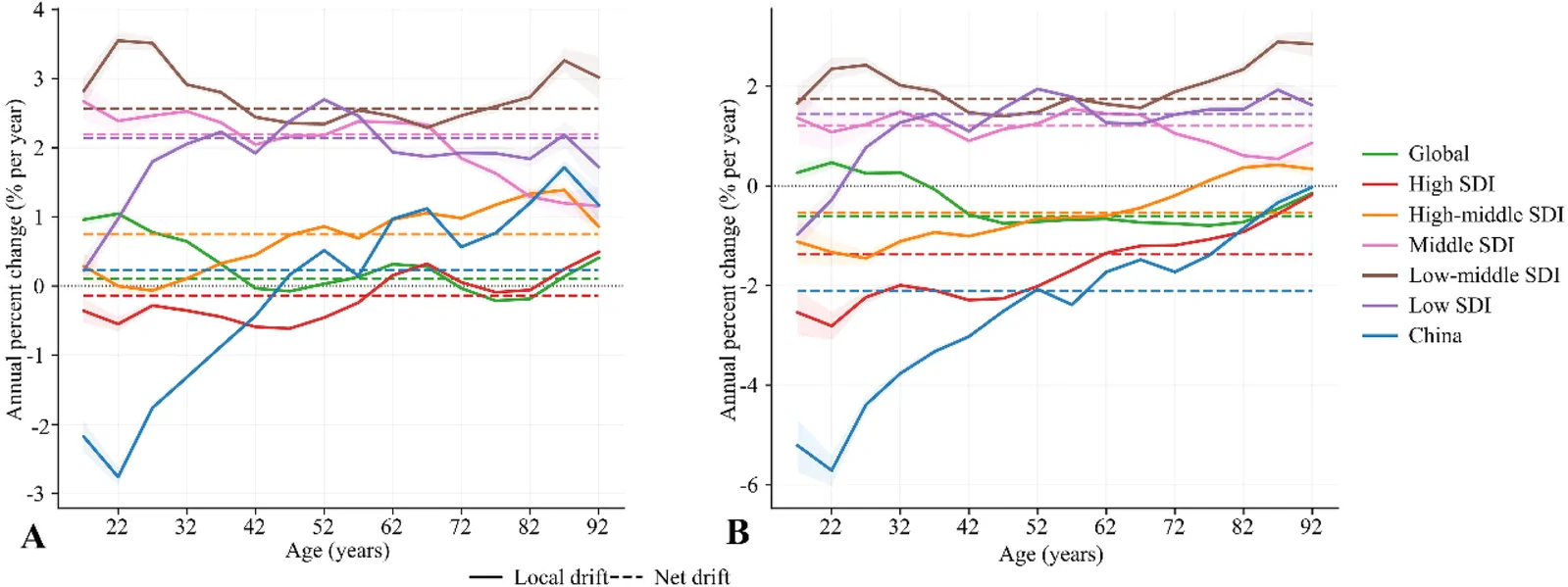
Net and local drift of female breast cancer incidence and mortality, 1990‒2019. (A) Incidence; (B) Mortality. Solid lines indicate local drift, and dashed lines indicate net drift.

## Discussion

Using GBD 2021 data, this study comparatively assessed China’s female breast cancer burden in relation to the global population and five SDI-level groups. It further examines key attributable risk profiles, sources of attributable mortality changes, and APC characteristics. The results showed that between 1990 and 2021, China’s female breast cancer ASIR showed a slight increase, while ASMR exhibited a consistent decline. By 2021, both ASIR and ASMR in China were lower than the global average and all five SDI tiers. In the attributable risk profile, high red meat consumption and secondhand smoke exposure remain prominent attributable risks in China, while metabolic risks such as high BMI and high fasting plasma glucose show increasing relative contributions. Four-effect decomposition indicates that population growth and age-structure change are primary contributors to the increase in attributable deaths, while declining age-specific mortality rates partly offset the net increase. APC analysis further reveals that China’s incidence net drift remains slightly positive, whereas the mortality net drift is negative, with incidence increases concentrated primarily among middle-aged and older women. Overall, China’s female breast cancer burden diverges from both the relatively mature control patterns in high-SDI regions and the synchronous rise in incidence and mortality observed in low to middle SDI regions. Instead, it exhibits transitional characteristics between these two patterns.

Across global and SDI-stratified comparisons, China demonstrates a divergent pattern featuring a gradual increase in incidence burden alongside declining ASMR. This finding is consistent with the heterogeneity in breast cancer burden documented in prior global studies. High-SDI regions typically show higher incidence levels and more pronounced mortality declines, whereas lower SDI regions continue to face the dual pressure of rising incidence and mortality burdens.^4^^,^^6^ The ASMR in China decreased significantly, which suggests an improving trend in the mortality burden of female breast cancer. Previous studies have suggested that advances in cancer detection and treatment, optimization of therapeutic approaches, and improved access to healthcare may have contributed to the reduction in breast cancer mortality. However, the GBD data used in this study do not allow us to separately assess the extent to which each of these factors contributed to the observed decline.^9^^,^^23^ Meanwhile, the ASIR exhibited a slight increase, likely reflecting changes in the incidence patterns of female breast cancer in China.^10^ As GBD data are population-level estimates, this study could not further distinguish the relative contributions of actual increases in incidence risk versus increased detection through diagnosis.^24^ However, considering China’s large population base and rapid aging process, even relatively moderate rate changes may translate into continuously growing case numbers and healthcare demands.^10^^,^^18^ Future breast cancer prevention and control should continue to focus on changes in disease burden. Evidence from screening strategies, risk factor management, and healthcare resource allocation should also be considered. The findings of this study provide epidemiological evidence to inform public health planning but cannot directly evaluate the effectiveness of specific interventions.^25^

The shift in attributable risk profiles indicates that traditional behavioral and environmental exposures remain important contributors. At the same time, the contribution of metabolic risks continues to increase, suggesting changes in the risk pattern of female breast cancer. Globally and across the SDI gradient, significant variations exist in the relative contributions of high BMI, high fasting plasma glucose, alcohol use, tobacco-related exposures, and dietary risks across regions at different development levels.^7^^,^^26^ This pattern may indicate that socioeconomic development, urbanization, lifestyle changes, and healthcare accessibility collectively shape the attributable risk structure for breast cancer.^7^ China exhibits distinct transitional characteristics within this gradient. On the one hand, high red meat consumption and secondhand smoke exposure remain ranked among the top attributable risks, and in 2021, the PAFs for both high red meat consumption and secondhand smoke exposure in China exceeded the global average. On the other hand, PAFs associated with high BMI and high fasting plasma glucose were higher than those in low-SDI and low-middle-SDI regions and approached those of certain higher SDI tiers. This indicates that the risk structure for female breast cancer in China is not a simple replacement of traditional risks by metabolic risks, because traditional behavioral and environmental risks persist while metabolic risks continue to rise.^7^^,^^13^

The rise in metabolic risks warrants particular attention. Previous studies in China have indicated the increasing importance of metabolic factors such as high BMI and high fasting plasma glucose in the attributable burden of female breast cancer.^11^^,^^12^ Previous studies have provided biological insights into the underlying mechanisms, obesity may contribute to breast cancer development through insulin resistance, chronic low-grade inflammation, increased estrogen biosynthesis, elevated circulating insulin and IGF-1 levels, and activation of pro-proliferative signaling pathways such as PI3K/AKT. These mechanisms are particularly relevant to estrogen receptor-positive breast cancer risk.^27^^,^^28^ However, this study does not directly validate these mechanisms. Rather, it demonstrates from a population-burden perspective that China’s breast cancer attributable risk profile is undergoing a metabolic shift. The increasing contribution of metabolic risks highlights their potential importance in future breast cancer prevention and control. These findings may provide a reference for strategies targeting weight management, dietary improvement, physical activity promotion, blood glucose control, and secondhand smoke exposure reduction. However, the effectiveness of these interventions requires further investigation.^29^

The four-effect decomposition further reveals the different sources underlying changes in attributable deaths. Unlike traditional trend analysis that only indicates increases or decreases in attributable deaths, this study integrates population growth, age-structure change, age-specific mortality rate changes, and PAF variation into a unified framework.^30^ This approach helps distinguish the relative contributions of demographic pressures, mortality risk changes, and risk attribution changes.^31^ The results indicate that the increase in attributable deaths in China primarily stems from population growth and age-structure change, whereas the overall contribution of PAF changes was relatively limited. Declining age-specific mortality rates partly offset the net increase in attributable deaths. This pattern suggests that the age-specific mortality risk for female breast cancer in China may have decreased, possibly reflecting improvements in diagnosis and comprehensive management.^4^ However, population growth and aging continue to amplify the absolute mortality burden. Compared with high-SDI regions, China still has potential for further improvement in the negative contribution resulting from the decline in age-specific mortality rates. Compared with low-middle- and low-SDI regions, China has demonstrated more significant improvements in mortality risk. China is therefore in a transitional phase characterized by concurrent improvement in mortality risk and increased absolute burden. The decline in ASMR reflects a favorable change in mortality risk. However, it does not necessarily indicate a parallel reduction in healthcare service pressure, particularly in the context of population growth and ongoing population aging.^32^^,^^33^

APC and drift analyses further demonstrate that changes in China’s female breast cancer burden stem not merely from age accumulation effects, but from the integrated influences of age, period, and birth cohort. Female breast cancer mortality risk in China escalates with advancing age, exhibiting stronger age-specific mortality effects in older population groups. Local drift results indicate that incidence growth is primarily concentrated among middle-aged and elderly women, while mortality local drift remained negative across all age groups, although the magnitude of decline attenuated markedly at older ages. This pattern is consistent with previous APC analyses of breast cancer mortality trends.^14^ Compared with high-SDI regions, China still faces challenges including a positive incidence net drift and insufficient mortality improvement in elderly age groups. Compared with low-middle- and low-SDI regions, China’s mortality net drift was negative, indicating an overall favorable temporal trend in mortality.^7^^,^^14^ Overall, the APC results revealed a transitional pattern of breast cancer in Chinese women, characterized by rising incidence, improving mortality, and pronounced age heterogeneity. The increasing incidence was mainly observed among middle-aged, elderly, and older women, highlighting distinct age-specific variations in disease burden. These findings provide epidemiological evidence for understanding changing breast cancer patterns and underscore the importance of considering age-related differences in future prevention and control strategies.^34^^,^^35^

This study has several limitations. First, GBD 2021 estimates are model-based and depend on the quality of cancer registry data, vital statistics, and modeling assumptions; therefore, uncertainty may vary across countries and SDI levels. Second, the GBD attribution framework includes only risk factors with sufficient evidence and available exposure data. Important factors such as reproductive history, hormonal exposure, genetic susceptibility, molecular subtype, occupational exposure, screening coverage, and treatment accessibility were not included. Thus, the attributable risk profile reported here should not be interpreted as a complete etiological landscape. Third, this study was based on national- and SDI-level aggregated data and therefore cannot infer individual-level associations or capture within-China disparities in screening, diagnosis, treatment, and healthcare access. Fourth, PAF estimates rely on predefined exposure-outcome relationships, theoretical minimum risk exposure levels, and assumptions regarding confounding control. Because risk factors may be correlated, risk-specific attributable burdens should not be interpreted as additive or fully preventable individual-level cases. Finally, because age, period, and cohort are linearly dependent, APC estimates depend on the model parameterization and reference constraints. Therefore, specific period- and cohort-effect estimates should be interpreted cautiously.

## Conclusion

Overall, from 1990 to 2021, female breast cancer in China showed a slow rise in incidence, a decline in the ASMR, and a concurrent transformation in attributable risk structure. China exhibits a distinct transitional nature within the global SDI gradient: improvements in mortality risk are evident, yet population growth, aging, and metabolic risk accumulation remain important considerations for the future absolute burden, underscore the continuous surveillance These findings underscore the importance of continued surveillance of breast cancer burden, the optimization of disease management systems, and attention to risk-factor exposures in priority populations. Future prevention and control strategies can integrate the findings of this study with evidence from screening-effectiveness evaluations, clinical research, and health-policy studies. This will help further explore comprehensive measures to reduce breast cancer burden among Chinese women.

## Ethics approval and consent to participate

This study was based on publicly available aggregated data from the Global Burden of Disease Study 2021. No identifiable individual-level data, human participants, or animal experiments were involved. Therefore, additional ethical approval and informed consent were not required.

## Consent for publication

Not applicable.

## Authors’ contributions

Yixin Si and Jialin Liu: Data curation; formal analysis; software, validation; visualization; writing-original draft. Liuqiyu Xu, Wenya Ye, Jiating Li and Yun Peng: Investigation; validation, resources; writing-review and editing. Zhen Hu: Conceptualization; methodology; supervision; project administration; writing-review and editing.

## Funding

This research received no specific grant from any funding agency in the public, commercial, or not-for-profit sectors.

## Data availability statement

The data used in this study were obtained from the publicly available GBD 2021 database. The relevant data can be accessed through the Global Health Data Exchange or the GBD Results Tool. Data generated or organized during the present analysis are available from the corresponding author upon reasonable request.

## Declaration of competing interest

The authors declare no conflicts of interest.
