# Supplementary material for: Comparative analysis of female breast cancer burden in China along the global sociodemographic gradient: risk attribution, decomposition, and age-period-cohort evidence from GBD 2021
Source: Clinics (Sao Paulo). 2026 Sep 30;81:101166. doi: 10.1016/j.clinsp.2026.101166 (PMC13657178; doi:10.1016/j.clinsp.2026.101166)
Supplement: Supplementary file 1 [file mmc1.docx]

**CLINICS-D-26-01253_Supplementary Material**

**Supplementary Table 1** Detailed four-effect decomposition results by risk factor and region.

| **Location** | **Risk** | **Attributable deaths in 1990** | **Attributable deaths in 2021** | **Change in attributable deaths** | **Population growth** | **Age structure** | **Age-specific death rate** | **PAF change** |
| --- | --- | --- | --- | --- | --- | --- | --- | --- |
| Global | Diet high in red meat | 446531.4 | 786036.7 | 339505.3 | 357808.6 | 106745.0 | -65477.8 | -59570.6 |
|  | High fasting plasma glucose | 193264.7 | 435173.8 | 241909.1 | 175840.4 | 57470.8 | -37425.8 | 46023.8 |
|  | Secondhand smoke | 269649.1 | 410706.0 | 141056.8 | 201854.1 | 46832.7 | -30286.0 | -77343.9 |
|  | High body-mass index | 194624.9 | 426676.5 | 232051.5 | 158697.1 | 90153.1 | -56589.6 | 39790.9 |
|  | Low physical activity | 83805.2 | 165406.0 | 81600.8 | 71161.1 | 26286.2 | -15627.0 | -219.5 |
|  | High alcohol use | 124298.5 | 132356.7 | 8058.2 | 66612.3 | 30044.1 | -11792.9 | -76805.3 |
|  | Smoking | 89387.6 | 97065.6 | 7677.9 | 63710.2 | 10015.1 | -11511.9 | -54535.6 |
| High SDI | Diet high in red meat | 332893.6 | 426203.1 | 93309.5 | 148326.0 | 89085.1 | -140489.0 | -3612.4 |
|  | High fasting plasma glucose | 137678.5 | 220896.1 | 83217.6 | 68967.2 | 44854.1 | -63986.6 | 33382.9 |
|  | Secondhand smoke | 162259.1 | 148888.4 | -13370.7 | 62233.2 | 28928.9 | -66029.4 | -38503.4 |
|  | High body-mass index | 169927.6 | 284295.1 | 114367.5 | 74229.9 | 75763.0 | -77475.1 | 41849.8 |
|  | Low physical activity | 62677.7 | 89729.6 | 27051.9 | 29526.1 | 21308.7 | -24934.8 | 1152.0 |
|  | High alcohol use | 113994.1 | 104166.7 | -9827.4 | 31727.0 | 27990.9 | -49623.6 | -19921.7 |
|  | Smoking | 76439.6 | 70076.0 | -6363.6 | 33950.9 | 8674.9 | -31492.5 | -17497.0 |
| High-middle SDI | Diet high in red meat | 54504.0 | 119191.0 | 64687.1 | 57726.4 | 22068.3 | -5155.6 | -9952.0 |
|  | High fasting plasma glucose | 28029.0 | 77574.9 | 49545.9 | 33518.9 | 14032.2 | -2872.8 | 4867.6 |
|  | Secondhand smoke | 49862.7 | 78246.8 | 28384.1 | 45458.6 | 15760.4 | -4553.4 | -28281.4 |
|  | High body-mass index | 14194.5 | 65774.6 | 51580.1 | 19769.7 | 18383.7 | -1311.9 | 14738.6 |
|  | Low physical activity | 9434.9 | 26468.0 | 17033.2 | 11413.4 | 5600.1 | -579.2 | 599.0 |
|  | High alcohol use | 5114.2 | 9439.4 | 4325.2 | 3646.8 | 2363.8 | -681.0 | -1004.6 |
|  | Smoking | 6213.5 | 9478.2 | 3264.6 | 6425.9 | 1608.1 | -502.4 | -4267.0 |
| Middle SDI | Diet high in red meat | 21269.0 | 78803.2 | 57534.2 | 31769.7 | 7187.0 | 17975.8 | 601.9 |
|  | High fasting plasma glucose | 9455.0 | 40176.0 | 30721.0 | 15266.7 | 4005.8 | 8582.7 | 2865.8 |
|  | Secondhand smoke | 20870.3 | 68972.8 | 48102.5 | 29341.7 | 6357.6 | 16673.7 | -4270.4 |
|  | High body-mass index | 5104.5 | 36541.9 | 31437.3 | 10319.1 | 5451.7 | 6478.9 | 9187.6 |
|  | Low physical activity | 4489.5 | 17795.4 | 13306.0 | 6968.2 | 1882.2 | 3878.7 | 576.9 |
|  | High alcohol use | 1499.4 | 4728.8 | 3229.4 | 1638.3 | 730.2 | 1175.7 | -314.8 |
|  | Smoking | 2450.2 | 5971.0 | 3520.7 | 3284.1 | 496.8 | 1652.1 | -1912.2 |
| Low-middle SDI | Diet high in red meat | 18330.4 | 82671.3 | 64340.9 | 30478.3 | 4478.1 | 23989.2 | 5395.2 |
|  | High fasting plasma glucose | 10086.6 | 53740.8 | 43654.2 | 18514.0 | 3586.0 | 14590.1 | 6964.1 |
|  | Secondhand smoke | 20091.0 | 58699.4 | 38608.4 | 26485.5 | 3868.4 | 20719.3 | -12464.9 |
|  | High body-mass index | 3237.3 | 26214.1 | 22976.8 | 7387.9 | 3483.9 | 5973.7 | 6131.2 |
|  | Low physical activity | 3908.377 | 17530.87 | 13622.49 | 6466.227 | 1570.191 | 5188 | 398.0711 |
|  | High alcohol use | 1104.824 | 3697.445 | 2592.621 | 1387.367 | 266.0981 | 1194.857 | -255.701 |
|  | Smoking | 2397.988 | 5930.593 | 3532.605 | 3077.322 | 417.9665 | 2266.698 | -2229.38 |
| Low SDI | Diet high in red meat | 18780.69 | 78322.24 | 59541.55 | 35623.9 | 1116.228 | 21940.98 | 860.4442 |
|  | High fasting plasma glucose | 7748.426 | 42165.26 | 34416.84 | 17156.83 | 629.6983 | 10700.65 | 5929.656 |
|  | Secondhand smoke | 16202.54 | 55414.42 | 39211.88 | 27711.25 | 806.3212 | 17116.03 | -6421.72 |
|  | High body-mass index | 1824.25 | 13136.97 | 11312.72 | 4761.291 | 281.8628 | 3412.408 | 2857.16 |
|  | Low physical activity | 3166.841 | 13629.46 | 10462.62 | 6108.307 | 299.3377 | 3698.67 | 356.3071 |
|  | High alcohol use | 2319.565 | 9789.217 | 7469.651 | 4272.524 | 244.6457 | 2738.017 | 214.4651 |
|  | Smoking | 1694.367 | 5291.915 | 3597.548 | 2826.551 | 46.59142 | 1718.001 | -993.595 |
| China | Diet high in red meat | 65622 | 94825.52 | 29203.52 | 44770.37 | 25538.31 | -42244.1 | 1138.975 |
|  | High fasting plasma glucose | 33442.23 | 46040.15 | 12597.92 | 22124.05 | 13739.1 | -20252.2 | -3013.06 |
|  | Secondhand smoke | 59755.75 | 72356.22 | 12600.47 | 37646.92 | 19332.69 | -36814.5 | -7564.65 |
|  | High body-mass index | 14177.05 | 36896.77 | 22719.72 | 10197.16 | 13519.34 | -12284 | 11287.19 |
|  | Low physical activity | 9078.667 | 15807.12 | 6728.455 | 6797.69 | 4991.781 | -5572.97 | 511.958 |
|  | High alcohol use | 5444.7 | 5753.635 | 308.936 | 1870.333 | 2623.333 | -3411.21 | -773.524 |
|  | Smoking | 5645.564 | 5890.281 | 244.7171 | 4148.498 | 1802.696 | -2798.45 | -2908.03 |

**Supplementary Table 2** Local drift of female breast cancer incidence and mortality by age group and location, 1990–2019.

| **Panel A Incidence local drift** | | | | | | | |
| --- | --- | --- | --- | --- | --- | --- | --- |
| **Age Group** | **China** | **Global** | **High SDI** | **High-middle SDI** | **Middle SDI** | **Low-middle SDI** | **Low SDI** |
| 15‒19 | -2.178 (-2.400 to -1.956) | 0.962 (0.872 to 1.052) | -0.359 (-0.532 to -0.186) | 0.289 (0.080 to 0.498) | 2.669 (2.403 to 2.935) | 2.823 (2.612 to 3.035) | 0.226 (0.034 to 0.418) |
| 20‒24 | -2.760 (-2.885 to -2.634) | 1.047 (0.990 to 1.105) | -0.549 (-0.652 to -0.445) | -0.001 (-0.128 to 0.127) | 2.388 (2.201 to 2.577) | 3.550 (3.415 to 3.685) | 0.974 (0.843 to 1.105) |
| 25‒29 | -1.762 (-1.828 to -1.696) | 0.777 (0.745 to 0.810) | -0.281 (-0.331 to -0.231) | -0.064 (-0.136 to 0.007) | 2.464 (2.358 to 2.571) | 3.514 (3.421 to 3.606) | 1.801 (1.715 to 1.888) |
| 30‒34 | -1.320 (-1.368 to -1.271) | 0.647 (0.626 to 0.668) | -0.354 (-0.385 to -0.323) | 0.108 (0.059 to 0.157) | 2.528 (2.458 to 2.598) | 2.914 (2.851 to 2.977) | 2.054 (1.997 to 2.112) |
| 35‒39 | -0.872 (-0.910 to -0.834) | 0.319 (0.303 to 0.334) | -0.444 (-0.466 to -0.423) | 0.328 (0.290 to 0.366) | 2.363 (2.311 to 2.416) | 2.802 (2.748 to 2.856) | 2.229 (2.178 to 2.280) |
| 40‒44 | -0.432 (-0.461 to -0.403) | -0.033 (-0.046 to -0.021) | -0.590 (-0.606 to -0.574) | 0.452 (0.421 to 0.482) | 2.047 (2.005 to 2.090) | 2.444 (2.398 to 2.491) | 1.923 (1.877 to 1.968) |
| 45‒49 | 0.162 (0.138 to 0.187) | -0.076 (-0.087 to -0.065) | -0.614 (-0.627 to -0.600) | 0.738 (0.710 to 0.765) | 2.167 (2.127 to 2.207) | 2.359 (2.314 to 2.404) | 2.384 (2.337 to 2.431) |
| 50‒54 | 0.516 (0.491 to 0.541) | 0.031 (0.021 to 0.042) | -0.457 (-0.469 to -0.444) | 0.863 (0.836 to 0.890) | 2.182 (2.140 to 2.224) | 2.342 (2.296 to 2.388) | 2.697 (2.648 to 2.746) |
| 55‒59 | 0.147 (0.119 to 0.174) | 0.129 (0.118 to 0.140) | -0.239 (-0.251 to -0.226) | 0.690 (0.660 to 0.719) | 2.381 (2.336 to 2.427) | 2.546 (2.497 to 2.595) | 2.460 (2.404 to 2.515) |
| 60‒64 | 0.972 (0.944 to 1.000) | 0.316 (0.305 to 0.326) | 0.154 (0.142 to 0.166) | 0.963 (0.933 to 0.994) | 2.367 (2.318 to 2.416) | 2.458 (2.406 to 2.511) | 1.937 (1.878 to 1.997) |
| 65‒69 | 1.121 (1.089 to 1.153) | 0.279 (0.268 to 0.290) | 0.322 (0.310 to 0.334) | 1.056 (1.022 to 1.090) | 2.335 (2.276 to 2.393) | 2.292 (2.232 to 2.353) | 1.873 (1.805 to 1.941) |
| 70‒74 | 0.566 (0.526 to 0.607) | -0.033 (-0.046 to -0.020) | 0.054 (0.040 to 0.068) | 0.982 (0.941 to 1.023) | 1.848 (1.777 to 1.919) | 2.467 (2.396 to 2.537) | 1.926 (1.850 to 2.003) |
| 75‒79 | 0.768 (0.716 to 0.819) | -0.212 (-0.226 to -0.198) | -0.089 (-0.104 to -0.074) | 1.176 (1.125 to 1.227) | 1.630 (1.539 to 1.721) | 2.597 (2.508 to 2.687) | 1.919 (1.818 to 2.020) |
| 80‒84 | 1.202 (1.132 to 1.273) | -0.184 (-0.200 to -0.168) | -0.059 (-0.076 to -0.041) | 1.335 (1.268 to 1.402) | 1.293 (1.176 to 1.410) | 2.734 (2.621 to 2.848) | 1.840 (1.719 to 1.961) |
| 85‒89 | 1.715 (1.610 to 1.820) | 0.137 (0.117 to 0.157) | 0.245 (0.224 to 0.266) | 1.388 (1.294 to 1.481) | 1.197 (1.036 to 1.358) | 3.262 (3.096 to 3.429) | 2.188 (2.000 to 2.377) |
| 90‒94 | 1.175 (0.950 to 1.400) | 0.407 (0.371 to 0.444) | 0.494 (0.456 to 0.532) | 0.862 (0.685 to 1.040) | 1.158 (0.873 to 1.444) | 3.019 (2.712 to 3.327) | 1.724 (1.393 to 2.056) |

| **Panel B Mortality local drift** | | | | | | | |
| --- | --- | --- | --- | --- | --- | --- | --- |
| **Age Group** | **China** | **Global** | **High SDI** | **High-middle SDI** | **Middle SDI** | **Low-middle SDI** | **Low SDI** |
| 15‒19 | -5.207 (-5.740 to -4.671) | 0.266 (0.092 to 0.440) | -2.538 (-2.997 to -2.077) | -1.118 (-1.557 to -0.678) | 1.362 (0.849 to 1.878) | 1.658 (1.312 to 2.006) | -0.978 (-1.280 to -0.675) |
| 20‒24 | -5.710 (-6.016 to -5.404) | 0.467 (0.355 to 0.580) | -2.810 (-3.091 to -2.528) | -1.328 (-1.594 to -1.062) | 1.080 (0.715 to 1.446) | 2.350 (2.129 to 2.573) | -0.278 (-0.484 to -0.071) |
| 25‒29 | -4.392 (-4.544 to -4.240) | 0.253 (0.188 to 0.318) | -2.234 (-2.365 to -2.103) | -1.452 (-1.596 to -1.307) | 1.237 (1.038 to 1.436) | 2.426 (2.276 to 2.576) | 0.771 (0.637 to 0.905) |
| 30‒34 | -3.764 (-3.863 to -3.665) | 0.267 (0.229 to 0.306) | -1.996 (-2.067 to -1.924) | -1.111 (-1.200 to -1.023) | 1.488 (1.371 to 1.605) | 2.018 (1.923 to 2.113) | 1.273 (1.189 to 1.356) |
| 35‒39 | -3.325 (-3.399 to -3.250) | -0.070 (-0.099 to -0.041) | -2.097 (-2.147 to -2.048) | -0.931 (-0.998 to -0.864) | 1.254 (1.169 to 1.339) | 1.903 (1.825 to 1.982) | 1.452 (1.381 to 1.523) |
| 40‒44 | -3.023 (-3.082 to -2.964) | -0.574 (-0.597 to -0.550) | -2.291 (-2.328 to -2.254) | -1.008 (-1.062 to -0.953) | 0.907 (0.839 to 0.974) | 1.472 (1.404 to 1.539) | 1.098 (1.035 to 1.160) |
| 45‒49 | -2.514 (-2.563 to -2.465) | -0.752 (-0.772 to -0.731) | -2.257 (-2.287 to -2.227) | -0.854 (-0.901 to -0.806) | 1.137 (1.075 to 1.199) | 1.401 (1.337 to 1.465) | 1.562 (1.499 to 1.626) |
| 50‒54 | -2.073 (-2.118 to -2.027) | -0.720 (-0.739 to -0.701) | -2.014 (-2.041 to -1.988) | -0.656 (-0.700 to -0.612) | 1.249 (1.188 to 1.309) | 1.482 (1.421 to 1.543) | 1.945 (1.883 to 2.008) |
| 55‒59 | -2.384 (-2.431 to -2.338) | -0.677 (-0.695 to -0.658) | -1.691 (-1.715 to -1.667) | -0.629 (-0.673 to -0.584) | 1.542 (1.480 to 1.604) | 1.763 (1.701 to 1.826) | 1.786 (1.718 to 1.854) |
| 60‒64 | -1.728 (-1.775 to -1.680) | -0.663 (-0.681 to -0.645) | -1.346 (-1.370 to -1.323) | -0.598 (-0.644 to -0.552) | 1.451 (1.385 to 1.516) | 1.642 (1.576 to 1.708) | 1.266 (1.195 to 1.338) |
| 65‒69 | -1.482 (-1.533 to -1.430) | -0.735 (-0.753 to -0.716) | -1.205 (-1.228 to -1.182) | -0.439 (-0.488 to -0.390) | 1.426 (1.350 to 1.502) | 1.566 (1.492 to 1.639) | 1.244 (1.165 to 1.323) |
| 70‒74 | -1.726 (-1.786 to -1.667) | -0.757 (-0.776 to -0.737) | -1.191 (-1.215 to -1.167) | -0.194 (-0.249 to -0.139) | 1.053 (0.967 to 1.139) | 1.889 (1.808 to 1.969) | 1.431 (1.347 to 1.515) |
| 75‒79 | -1.395 (-1.463 to -1.326) | -0.795 (-0.816 to -0.774) | -1.071 (-1.095 to -1.047) | 0.110 (0.047 to 0.173) | 0.869 (0.766 to 0.971) | 2.100 (2.003 to 2.196) | 1.534 (1.429 to 1.639) |
| 80‒84 | -0.852 (-0.937 to -0.768) | -0.727 (-0.750 to -0.705) | -0.923 (-0.948 to -0.898) | 0.371 (0.295 to 0.446) | 0.610 (0.488 to 0.731) | 2.343 (2.230 to 2.456) | 1.537 (1.420 to 1.654) |
| 85‒89 | -0.333 (-0.452 to -0.213) | -0.459 (-0.485 to -0.432) | -0.562 (-0.591 to -0.533) | 0.422 (0.323 to 0.522) | 0.543 (0.385 to 0.702) | 2.894 (2.736 to 3.051) | 1.927 (1.754 to 2.100) |
| 90‒94 | -0.024 (-0.227 to 0.180) | -0.146 (-0.183 to -0.109) | -0.174 (-0.213 to -0.134) | 0.340 (0.185 to 0.496) | 0.866 (0.627 to 1.106) | 2.850 (2.596 to 3.105) | 1.620 (1.351 to 1.890) |

Note: Values are local drift percentages with 95% CIs. Local drift represents the annual percentage change in age-specific incidence estimated from the APC model. CI, Confidence Interval; SDI, Socio-Demographic Index.

**Supplementary Figure 1** Ranking changes in Population Attributable Fractions (PAFs) for female breast cancer risk factors across SDI regions, 1990 and 2021. Panels: (A) High SDI, (B) High-middle SDI, (C) Low SDI, (D) Low-middle SDI, and (E) Middle SDI. Green indicates behavioral risks, and purple indicates metabolic risks.

**Supplementary Figure 2** Age-Period-Cohort (APC) results for female breast cancer burden, 1990–2021. (A) Incidence; (B) deaths. Each panel shows age, period, and cohort effects. RR, Relative Risk; SDI, Socio-Demographic Index.
